# Implementation of WHO multimodal hand hygiene (HH) improvement strategy to reduce healthcare-associated infections (HAI) and VAP (ventilator-associated pneumonia) caused by multi-drug resistant acinetobacter baumanii (MDRAB) at Siloam Hospitals Surabaya (SHBS), Indonesia

**DOI:** 10.1186/2047-2994-4-S1-O18

**Published:** 2015-06-16

**Authors:** B Indah Mustikawati, N Syitharini, S Widyaningtyastuti, L Gunawan

**Affiliations:** 1 Siloam Hospitals, Surabaya, Indonesia

## Introduction

SHSB is a 160 bed private hospital in Surabaya, Indonesia. The average length of stay (ALOS) is 9.52 d. In 2010, HAI rates at SHSB were high (3.12%) and HH compliance among healthcare workers (HCW) was low (73.34%). At the same time, SHSB had MDRAB outbreak among ICU patients with VAP rate of 10.99/device-days. Therefore, there was a need for quality improvement to reduce HAI and VAP caused by MDRAB.

## Methods

The WHO strategy was implemented in 2010 in all departments and included: 1) *System change initiatives*: HH policy review and ward infrastructure survey q 6 m, dedciated budget for HH agents, evaluation of HCW tolerability and acceptability of alcohol-based handrub (ABHR) 2) *Training and education*: regular mandatory training for all HCW, doctor’s forum and antibiotic stewardship case study, e-learning, etc... 3) *Evaluation and feedback*: monthly HH compliance audit and reporting to governing body, monthly feedback, external audits. 4) *Reminders in the workplace*: posters in public and point of care areas, reminders via paging system, fingerprint attendance machine, etc.... 5) *Institutional Safety Climate*: CEO support and commitment, QI program, HH as key performance indicator, patient speak-up token in reminding doctors to perform hand hygiene.

## Results

We conducted monthly HH audit compliance since 2010 with total number of 31267 opportunities during 2233 observation sessions. The overall hospital-wide compliance with HH increased significantly from 73% in 2010 to 88% in 2014 (p<0.0001). The overall consumption of ABHR increased in parallel from 16.8 L in 2010 to 34.55 L per 1000 patient-days in 2014 (p=0.002). HAIs rate decreased from 3.12 (2010) to 0.10 (2014) per 100 admission (p=0.02). VAP caused by MDRAB decreased from 10.99/devices days (2010) to 0% (2014) per 1000 device-days (p=0.02). Hospital ALOS decreased from 9.52 in 2010 to 4.38 in 2014.

## Conclusion

Implementation of WHO Multimodal HH Improvement Strategy that is supported and committed by CEO and all hospital staffs, increased HCW compliance and subsequently reduced HAIs hospital-wide and VAP caused by MDRAB in ICU.

## Disclosure of interest

None declared.

